# Transient Signals and Inattentional Blindness in a Multi-object Tracking Task

**DOI:** 10.1177/2041669518754595

**Published:** 2018-01-19

**Authors:** Dakota B. Palmer, Yusuke Yamani, Taylor L. Bobrow, Nicole D. Karpinsky, Dean J. Krusienski

**Affiliations:** Department of Psychology, 6042 Old Dominion University, VA, USA; Department of Electrical & Computer Engineering, 6042 Old Dominion University, VA, USA; Department of Psychology, 6042 Old Dominion University, VA, USA; Department of Electrical & Computer Engineering, 6042 Old Dominion University, VA, USA

**Keywords:** inattentional blindness, visual awareness, salience

## Abstract

Inattentional blindness is a failure to notice an unexpected event when attention is directed elsewhere. The current study examined participants’ awareness of an unexpected object that maintained luminance contrast, switched the luminance once, or repetitively flashed. One hundred twenty participants performed a dynamic tracking task on a computer monitor for which they were instructed to count the number of movement deflections of an attended set of objects while ignoring other objects. On the critical trial, an unexpected cross that did not change its luminance (control condition), switched its luminance once (switch condition), or repetitively flashed (flash condition) traveled across the stimulus display. Participants noticed the unexpected cross more frequently when the luminance feature matched their attention set than when it did not match. Unexpectedly, however, a proportion of the participants who noticed the cross in the switch and flash conditions were statistically comparable. The results suggest that an unexpected object with even a single luminance change can break inattentional blindness in a multi-object tracking task.

Inattentional blindness (IB) occurs when an observer fails to notice conspicuous events or objects when their attention is directed elsewhere (Chabris, Weinberger, Fontaine, & Simons, 2011; Mack & Rock, 1998; Most, Scholl, Clifford, & Simons, 2005; Most, Scholl, Jimenez, Clifford, & Chabris, 2001; Simons & Chabris, 1999; Simons, 2000). IB can arise even with a direct fixation at an unexpected object (Drew, Võ, & Wolfe, 2013; Memmert, 2006), which suggests that IB arises not due to lack of direct fixations but from postsensory processes. Even expert observers and professional practitioners (Drew et al., 2013; Memmert, 2006), as well as those who are familiar with the phenomenon (Simons, 2010), are not immune to the effect of IB. In the real world, IB can lead to critical failures and have disastrous outcomes in professional tasks, such as piloting a vehicle (Fischer, Haines, & Price, 1980) or screening medical scans for abnormalities (Potchen, 2006). Thus, understanding the mechanisms underlying IB may help visual display designers for transportation and health-care systems select effective visual alerts that break operators’ IB.

Experimentally, Mack and Rock (1998) demonstrated IB in their static IB paradigm. In their experiment, observers were asked to judge which arm of a cross that was briefly presented at the center of the display was shorter. In a critical trial, an unexpected object appeared simultaneously with the cross, and observers reported whether they saw anything unusual after the completion of the trial. Approximately 25% of the observers failed to report the presence of the unexpected object, showing IB even in a simple perceptual task. Surprisingly, color changes and movements of the unexpected object did not entirely eliminate IB (Mack & Rock, 1998), suggesting that object saliency itself does not always capture observers’ attention (see Simons, 2000 for review).

More recently, Most et al. (2001; also 2005) showed that not only do bottom-up factors such as saliency of an unexpected object influence IB, but top-down factors such as attention sets influence IB in a dynamic multi-object tracking task. Participants were asked to view a dynamic visual display consisting of white and black circles and squares moving on random trajectories and to count the number of times either white or black objects deflected off the side of the display boundary while fixating at the center point of the display. Following two standard trials, on the third critical trial, an unexpected object which was visible for 5 seconds entered the display from the right side and traveled on a straight path through the center of the screen, exiting on the left side. The features of the unexpected *critical stimulus* such as shape, color, and luminance were systematically manipulated in a series of their experiments. After the critical trial, participants were asked if they noticed *anything unusual* and, if so, to describe what they saw. Depending on the unexpected object’s properties and their attention sets, as many as 100% of participants failed to notice it. Note that these participants detected the critical object in the following *full attention* trial, where they were asked to fixate on the display center without performing the tracking task. Strikingly, Most et al.’s (2001) showed that the probability of noticing an unexpected object depends on similarity between visual features of an unexpected object and those in participants’ attention set.

Thus, Most et al. (2001) showed that the probability of noticing unexpected objects depends on the degree of correspondence between an observer’s attention set and the stimulus property of an unexpected object. However, whether a highly salient, unexpected object overrides the top-down influence on attention and allows observers to notice the object remains unclear. Previous research indicates that highly salient and unexpected events do not necessarily promote visual awareness while performing a primary monitoring task (Simons & Chabris, 1999). Extending Mack and Rock’s (1998) study, Simons and Chabris (1999) asked participants to watch videos showing two teams of three players in black or white shirts (black vs. white teams) and silently count the number of passes made by one of two teams. In the middle of the tape, an experimenter wearing a gorilla costume (in black) entered from the left side of the action, thumped its chest, and then continued across the field of view. Results not only indicated that roughly half of participants failed to detect the Gorilla but also corroborated the attention set account of IB (Most et al., 2001) in that twice more number of participants noticed the Gorilla in the black condition than the white condition. Such results highlight the possibility that saliency of an unexpected event influences visual awareness more strongly when properties of the event match with participants’ attention set.

The current study aims to replicate the findings of Most et al. (2001) and examine the effects of levels of object saliency on visual awareness in a dynamic multi-object tracking task. We manipulated observers’ attention set (track black or white objects while ignoring white or black objects, respectively) and salience of an unexpected moving object (constant, switch, or flash). The unexpected object did not change its luminance (the constant condition), switched its luminance once (the switch condition), or flashed repetitively (the flash condition) in the tasks. We predicted that participants would notice the unexpected object more frequently in the flash condition than the switch condition, because the repetitive flashes create more frequent transient signals than the single switch. Furthermore, we predicted that participants would notice the unexpected object more frequently in the switch condition than the constant condition.

## Method

### Participants

One hundred twenty participants (96 females; mean age = 21.17 years, *SD* = 5.81; mean near visual acuity = 20/22.75; mean far visual acuity = 20/21.27) were recruited from the community of Old Dominion University. This study was reviewed and approved by the institutional review board at Old Dominion University. All participants were screened for normal or corrected-to-normal vision and normal color perception. They received a research participation credit for their participation.

### Stimuli

Following Most et al.’s (2001) methodology, each stimulus display contained four circle and four square objects on a gray background (luminance = 32.1 cd/m^2^) with a blue fixation point at the center of the display. One group of objects was colored black (luminance = 1.5 cd/m^2^) while the other was colored white (luminance = 88.0 cd/m^2^).

**Figure 1.**
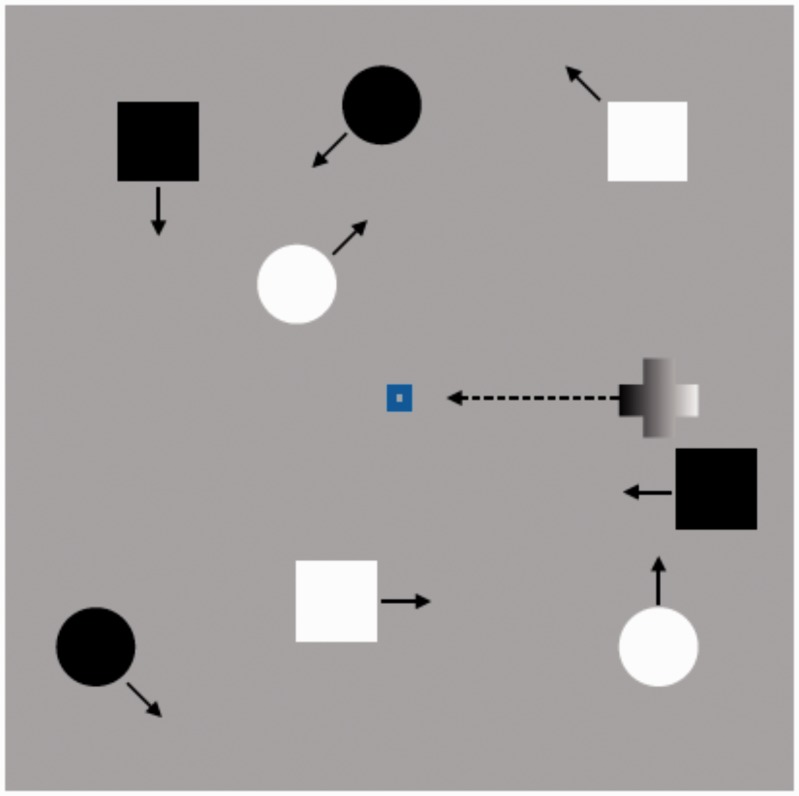
An example of the visual display in a critical event trial. Circle and square objects move randomly while an unexpected object flickering at 7.5 Hz enters from the right side of the display, over the middle fixation point, and exits the left side of the display.

Figure 1 illustrates a sample of the stimulus display. During the task, the objects moved across random paths at a variable rate ranging from 2° to 5° per second. The moving objects deflected at the display boundaries such that the angle of reflection was equal to the angle of incidence with the boundary.

In the critical trials, a cross with the same horizontal and vertical dimensions as circles and squares (1.0° × 1.0°) moved horizontally and linearly across the center of the screen in front of the fixation point from right to left. The luminance value of the cross remained the same (control condition), changed from white to black or black to white (switch condition), or rapidly alternated between black and white (flashing condition; frequency = 7.5 Hz, mean luminance = 14.1 cd/m^2^) through its path. Each of precritical and critical trials lasted 15 seconds.

### Procedure

Participants were randomly assigned to one of the three experimental conditions: Control, Switch, or Flashing. Within each condition, half of the participants attended to black objects while the others attended to white objects. Participants were instructed to maintain gaze on the fixation point, attend to target objects (black vs. white), and count the number of times target objects deflected off the display boundaries. Although participants were instructed to maintain their gaze on the central fixation point while completing the tracking task, we did not use eye-tracking to ensure this was the case, as IB is shown to arise even when observers directly fixate at unexpected objects (e.g., Drew et al., 2013; Mack & Rock, 1998).

Each trial began with the objects at random initial positions and central fixation point appearing in the visual display and motionless for 1 second. The objects then moved throughout the visual display until the trial ended. Participants completed a sequence of six experimental trials. The fifth and sixth trials contained the critical object. Five seconds after the beginning of the fifth and sixth trials, the critical cross entered and was visible for 5 seconds.

At the end of each critical trial, participants were asked to report whether an unusual event occurred during the trial to gauge their awareness of the critical event. Pressing “1” on the numeric keypad indicated that they detected an unusual event (presumably the cross) and “2” otherwise. If participants indicated that something unusual occurred, they were asked to report a description of the unusual event they perceived.

The sixth trial was a full attention trial, in which participants were instructed to passively view the display while fixating on the central fixation point, rather than counting the number of times objects deflected off the display boundaries. The full attention trial was performed to ensure that participants were able to perceive the object when attention was not diverted. Thus, data from those who failed to observe the critical object on the sixth trial were removed from the analysis. At the end of all trials except for the full attention trial, participants were asked to report the total number of times target objects deflected off the edges of the display by typing the number using the numeric keypad when prompted. Participants then received feedback regarding accuracy in the counting task after each trial for 750 milliseconds, consisting of *correct* or *incorrect* with the actual correct number of deflections.

### Statistical Analyses

Instead of the standard null hypothesis significance tests, we employed default Bayesian tests (Rouder, Speckman, Sun, Morey, & Iverson, 2009) using BayesFactor package in R (Morey, Rouder, & Jamil, 2015). The default Bayesian tests can provide evidence for the null hypothesis and Bayes factors provide a meaningful measure of evidence strength. The measure of evidence strength is *B*_10_, denoting the ratio of the likelihood that data support an alternative model that includes an effect of interest to the likelihood that data support the null model that excludes the effect. For example, a Bayes factor of 3 indicates that an alternative model is 3 times more likely resulting from the data than the null model, while a Bayes factor of 1/3 indicates substantial evidence for the null model over an alternative model. Descriptive terms for each comparison come from Jeffreys (1961) and Wetzels et al. (2011).

## Results and Discussion

Data from 15 participants were removed from the analysis due to failure to report the unexpected cross in the full attention trial. Table 1 presents the number of participants who noticed the unexpected cross across the three conditions. Omnibus analysis showed comparable noticing rates across the three experimental conditions, *χ*^2^(2)* = *3.46, *B*_10_ = 1/5.40. However, the results show that participants from the control condition noticed the unexpected cross more frequently when the luminance of the cross matched with their attention set, replicating Most et al. (2001). When attending to black objects, all the participants noticed the black cross while 57% of them noticed the white cross, and the data gave substantial evidence for a difference, *χ*^2^(1)* = *2.35, *B*_10_ = 4.85. Similarly, when attending to white objects, all the participants noticed the white cross while 42% of them noticed the black cross, which again provided strong evidence for a difference, *χ*^2^(1)* = *3.15, *B*_10_ = 7.80.

**Table 1.** Number of Participants on the Three Experimental Conditions Who Noticed the Unexpected Cross in the Critical and Full-Attention Attention Trials.

Unexpected object	Attention set	*n*	Trial type	
			Critical	Full attention
		Control		
Black objects	White objects	10	5	7
	Black objects	10	10	9
White objects	White objects	10	9	7
	Black objects	10	5	7
		Switch		
Black to White	White objects	10	7	9
	Black objects	10	9	10
White to Black	White objects	10	9	9
	Black objects	10	9	10
		Flashing		
Flashing	White objects	20	18	19
	Black objects	20	19	18

Contrary to our expectation, the single transient signal of the unexpected luminance-switching cross produced rates of response comparable to those with the highly salient flashing cross (86% vs. 91% for the switch and flash conditions, respectively), *χ*^2^(1)* = *.50, *B*_10_ = 1/4.50. Furthermore, the data also gave substantial evidence that target type (white or black) does not influence rates of noticing (*B*_10_ = 1/3.16 and *B*_10_ = 1/3.96 for the switch and flash conditions, respectively). Within the switch condition, the data produced substantial evidence against the effect of the order of the luminance change (white to black vs. black to white) on rates of noticing, *χ*^2^(1)* = *.23, *B*_10_ = 1/3.38.

To compare between the control and switch conditions more directly, we compared the proportions of responders when the luminance of the cross matched with their attention set (the match condition) and when it did not (the mismatch condition) in the control condition separately to the proportion of responders in the switch condition. Data showed anecdotal trends in favor of the null when comparing the match condition to the switch condition, *χ*^2^(1)* = *.91, *B*_10_ = 1/2.02. However, data provided strong evidence that the switch condition produced a greater noticing rate than the mismatch condition, *χ*^2^(1)* =*5.88, *B*_10_ = 10.85.

There are three points to highlight from the results. First, using Most et al.’s (2001) multi-object tracking paradigm, we replicated the effect of attention set on visual awareness. Compared to the proportions of participants reported in Most et al. (2001, Experiment 1), the effect of attention set was, though statistically reliable, markedly smaller in the current study. The smaller number of participants in the control condition in the current study (*N* = 31), as opposed to Most et al.’s (2001) study (*N* = 145), could have contributed to the discrepancy between the two studies.

Second, the results imply that the participants noticed the cross because luminance of the object matched with the attention set for a half of the time during the critical trial in the switch and the flash conditions. Participants attending to white objects, for example, might have noticed the cross on the switch or flash condition because the luminance of the cross was white half of the time that the critical display contained the cross. Future research should investigate the relationship between visual awareness and the influence of the amount of time that features of an unexpected target match with those in participants’ attention set when the features of the target vary dynamically in a multi-object tracking task. One alternative account of the current results is that transient signals captured attention. Thus, additional research can directly investigate the effects of transient signals of an unexpected object by manipulating the number of transient signals on IB while controlling the amount of time that the luminance of the unexpected object matches with participants’ attention set.

Finally, it should be noted that the current results are not likely due to an inadequate sample size or a ceiling effect. The experiments reported in Most et al. (2005) used 20.5 participants in each group on average, which is comparable to the current study. Furthermore, the counting accuracy data showed that participants’ tracking performance was substantially poorer in the flash condition than the control condition, independent-samples *t*(70) = 2.45, *B*_10_ = 3.07, and that performance in the switch condition was statistically equivalent to that in the control condition (*B*_10_ = 1/4.18), indicating that the switch and flash conditions were no easier than the control condition. Nonetheless, future research may reduce noticing rates by making the unexpected object smaller and move faster.
